# Assessment of Brainstem Function with Auricular Branch of Vagus Nerve Stimulation in Parkinson’s Disease

**DOI:** 10.1371/journal.pone.0120786

**Published:** 2015-04-07

**Authors:** David Weise, Melanie Adamidis, Fabio Pizzolato, Jost-Julian Rumpf, Christopher Fricke, Joseph Classen

**Affiliations:** 1 Department of Neurology, University of Leipzig, Leipzig, Germany; 2 Department of Neurological, Neuropsychological, Morphological and Movement Sciences, University of Verona, Verona, Italy; University of Toronto, CANADA

## Abstract

**Background:**

The efferent dorsal motor nucleus of the vagal nuclei complex may degenerate early in the course of Parkinson’s disease (PD), while efferent nucleus ambiguous, the principal source of parasympathetic vagal neurons innervating the heart, and afferent somatosensory nuclei remain intact.

**Objective:**

To obtain neurophysiological evidence related to this pattern, we tested processing of afferent sensory information transmitted via the auricular branch of the vagus nerve (ABVN) which is known to be connected to autonomic regulation of cardiac rhythm.

**Methods:**

In this cross-sectional observational study, we recorded (i) somatosensory evoked potentials (ABVN-SEP) and (ii) cutaneo-cardioautonomic response elicited by stimulation of the ABVN (modulation of heart-rate variability (HRV index; low frequency power, ln(LF), high frequency power, ln(HF); ln(LF/HF) ratio)) in 50 PD patients and 50 age and sex matched healthy controls. Additionally, auditory evoked potentials and trigeminal nerve SEP were assessed.

**Results:**

Neither ABVN-SEP nor any of the other functional brainstem parameters differed between patients and controls. Although HRV index was decreased in PD patients, modulation of ln(LF/HF) by ABVN-stimulation, likely indicating cardiac parasympathetic activation, did not differ between both groups.

**Conclusions:**

Findings do not point to prominent dysfunction of processing afferent information from ABVN and its connected parasympathetic cardiac pathway in PD. They are consistent with the known pattern of degeneration of the vagal nuclei complex of the brainstem.

## Introduction

**Fig 1:**
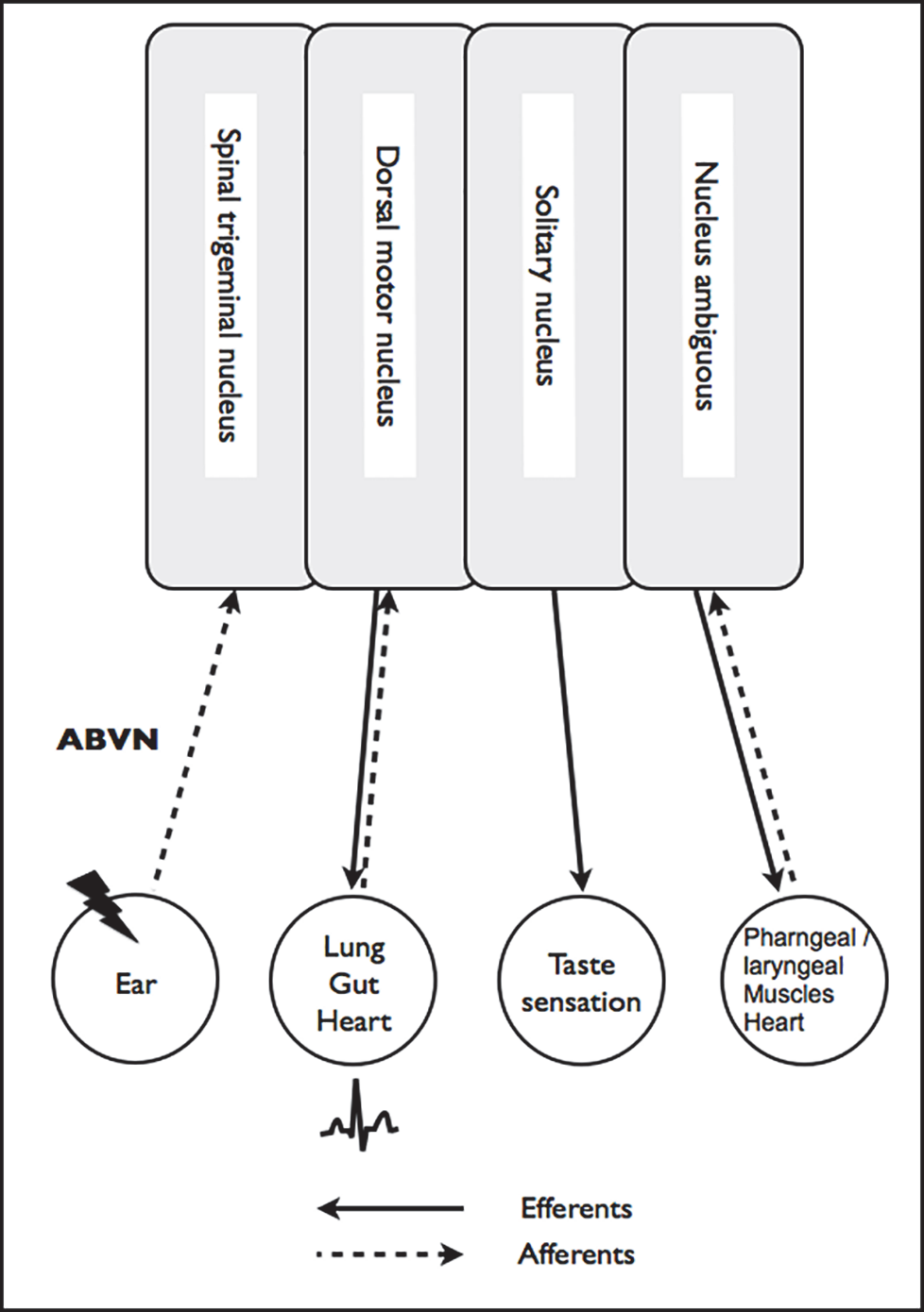
Schematic illustration of the vagal nuclei complex and its afferents and efferents.

Although Parkinson’s disease (PD) presents principally as a movement disorder and is mainly characterized by degeneration of dopaminergic neurons in the substantia nigra pars compacta, it has been claimed that other brainstem nuclei such as the dorsal motor nucleus (DMN) of the vagus nerve (VN) may be involved early in the course of the disease [1]. The efferent DMN is part of the vagal nuclei complex (Fig. 1). This complex additionally comprises the efferent nucleus ambiguous and afferent nuclei, namely the solitary nucleus. The VN also includes axons which converge onto the spinal trigeminal nucleus [2]. The DMN mainly innervates enteric neurons. In contrast, cardiac function is controlled by the nucleus ambiguous and, with possibly smaller contributions, by the DMN, with differential functional topography of left and right brainstem vagal nuclei [3,4].

Neuronal degeneration in PD is believed to affect the DMN [1,5] and, in part, the visceroafferent solitary nucleus [6], whereas the nucleus ambiguous and the somatosensory nuclei are spared [6,7]. From a clinical point of view, degeneration of the DMN may in part be responsible for autonomic, gastrointestinal, but also cardiac dysfunction, symptoms that often already occur in a premotor period of PD [8].

One way of addressing integrity of the vagal nuclei complex is by recording somatosensory evoked potentials (SEP) after stimulation of the auricular branch of vagus nerve (ABVN, ABVN-SEP, [9]). On electric stimulation of ABVN in the external auditory meatus, a specific neuronal response can be recorded as bipolar evoked far field potentials at the scalp [9–13]. ABVN-SEP potentials occur at millisecond latencies similar to early auditory evoked potentials (AEP), indicating that they may originate from brainstem vagal nuclei [9]. Patients with Alzheimer’s disease (AD) showed prolonged latencies [13], whereas patients with vascular dementia did not [12]. These results are in line with degeneration of the vagal nuclei complex in the course of AD [14]. Given the above mentioned pattern of degeneration in PD, sparing of the somatosensory nuclei would predict that ABVN-SEP should remain unaffected in PD, a conclusion challenged by a single pilot study from our lab [11].

Animal and human studies have revealed evidence for anatomic and functional cutaneo-cardioautonomic connections, mediated by the ABVN [15–19]. We, therefore, rationed that ABVN stimulation may also offer an opportunity to probe autonomic functions of the vagal nuclei complex in PD, apart from providing insight into brainstem processing of circumscribed somatosensory afferents.

Thus, recording of ABVN-SEP combined with ABVN-mediated cardiac autonomic modulation allowed us to address the following two questions: Are somatosensory efferents part of the degeneration affecting the vagal nuclei complex and are cutaneo-cardioautonomic reflexes compromised in PD? The answers to these questions may provide important information with relevance to the extent and functional consequences of the degeneration of the vagal nuclei complex pathology in PD.

## Subjects and Methods

### Standard protocol approvals, registrations, and patient consents

The study was approved by the Ethics Committee of the Medical Faculty of the University of Leipzig (reference no.: 232-09-28092009) and all participants gave their written informed consent.

### Demographic and clinical data

A population of 50 patients with a clinical diagnosis of PD according to the British brain bank criteria [20] was recruited from patients treated at the Department of Neurology of the University of Leipzig. Exclusion criteria were deep brain stimulation, a clinical history of stroke or traumatic brain injury. All patients, except one, were on antiparkinsonian medication. Fifty healthy, age and sex matched subjects with normal results on neurological examination were recruited as a healthy control group (CTRL). Exclusion criteria for CTRL were history of stroke, traumatic brain injury or neurodegenerative disease, and clinical signs of PD. The Montreal Cognitive Assessment (MoCa) was used for assessment of cognition [21].

### Auricular branch of vagus nerve somatosensory evoked potentials (ABVN-SEP)

Assessment of the ABVN-SEP was done as reported by Fallgatter and co-workers [10,9]. In brief, for transcutaneous electric stimulation of the left and right ABVN the stimulation electrodes (custom-made fine silver wires) were attached to the skin of the inner side of the tragus at the outer ventral edge of the external auditory meatus. ABVN-SEP were elicited using electric stimuli (electric square impulses of 0.1 ms duration, stimulation intensity 8 mA, frequency 0.5 Hz) on each side.

**Fig 2:**
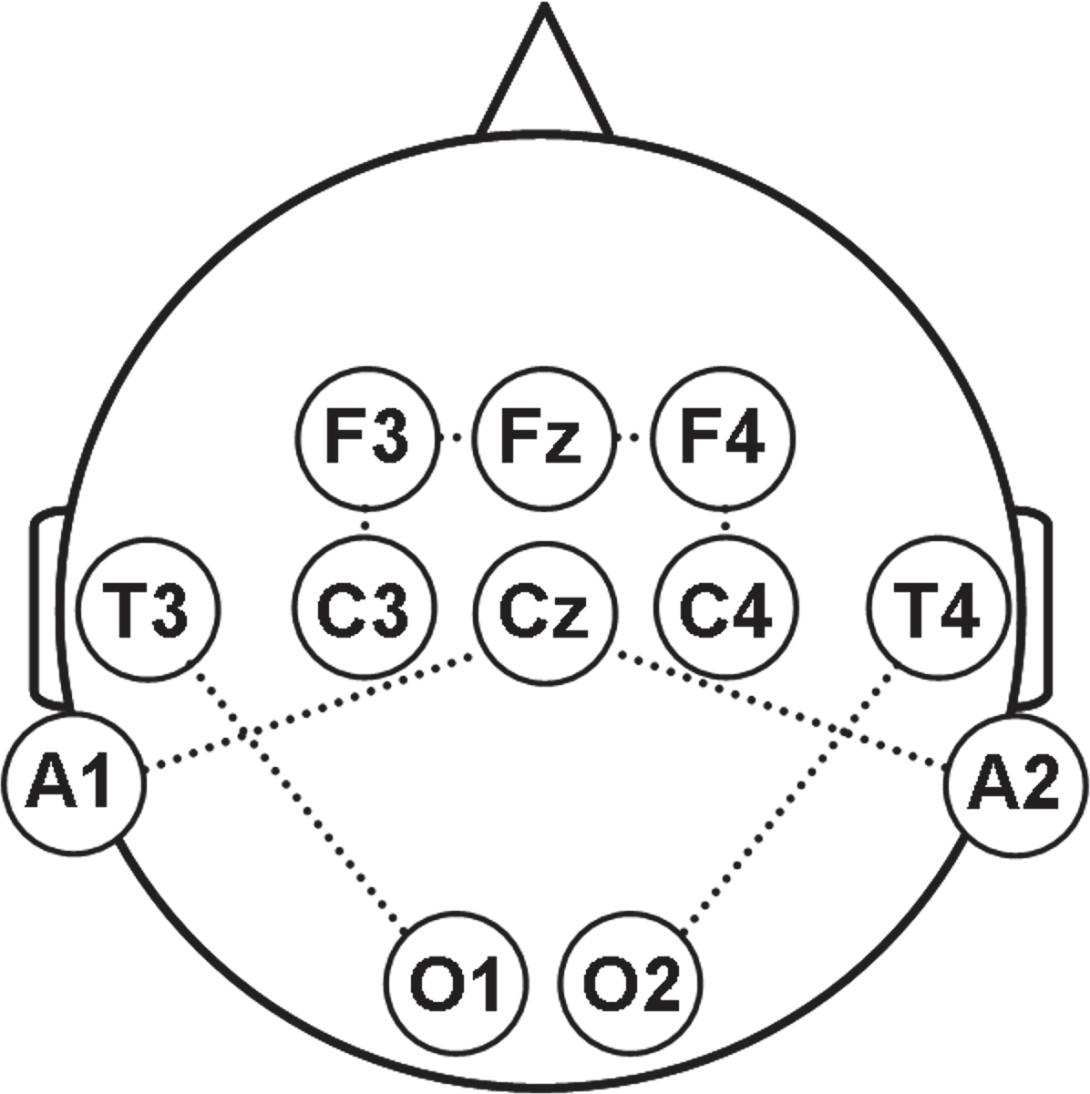
Schematic illustration of bipolar recordings from the scalp (10–20 system).

Evoked potentials were recorded bipolarly from Fz–F3, C3-F3, O1-T3 and Cz-A1 (left ABVN), and Fz–F4, C4-F4, O2-T4 and Cz-A2 (right ABVN) according to the international 10–20 system (Fig. 2). One hundred artifact-free epochs (band-pass filter 0.1 Hz −1 kHz, artifact criterion ± 40 μV, analysis time 20 ms), were collected and averaged using a conventional EMG/EP-system (NeuroConn, Germany) at least two times. Peak latencies (P1, N1, P2) and peak-to-peak latencies of early components (P1-N1, N1-P2) and peak-to-peak amplitudes (P1-N1, N1-P2) were determined semi-automatically and used for further statistical analyses. The analysis was performed offline with the investigator (D.W.) blinded for the identity and clinical status of the participants.

### Auditory evoked potentials (AEP) and trigeminal nerve somatosensory evoked potentials (TrigN-SEP)

AEP were generated by a click stimulus (minimum 2 * 500 clicks on each side, stimulation duration 0.1 ms, frequency 15 Hz, intensity 80 dB) via headphones and recorded bipolarly from A1-Cz (left) and A2-Cz (right), respectively. TrigN-SEP were recorded bipolarly from C5-Fz (left) and C6-Fz (right) and generated by transcutaneous electric stimulation of the trigeminal nerve at the upper and lower lip with the same stimulation electrode used for ABVN-SEP (minimum 2 times 50 stimuli on each side of 0.2 ms duration, frequency 2.3 Hz, intensity 5–6 mA). Peak latencies of the early brainstem components of the AEP (I–V) and cortical components of the TrigN-SEP (N13, P19, N27) were determined semi-automatically and used for further statistical analyses.

### Heart rate variability (HRV)

After at least 5 min of rest, RR intervals were measured on electrocardiograms at normal breathing for 3 min under resting condition and for around 3 min during left or right transcutaneous ABVN stimulation (while ABVN-SEP were recorded) each in a pseudorandomized order via the computer-based system ProScicard (Medset Medizintechnik GmbH, Hamburg, Germany; [22]). Several indices of the heart rate variability (HRV) were automatically computed: number of all RR-intervals / maximal frequency (HRV index, higher index indicates higher variability), power spectral analysis in the low (ln(LF), 0.05–0.15 Hz) and in the high frequency spectrum (ln(HF), 0.15–0.5 Hz) and the low frequency/high frequency ratio (ln(LF/HF) ratio). The HRV index and the ln(LF/HF) ratio at rest were considered as a marker of sympatho–vagal interaction [22,23].

### Statistical Analysis

Since Kolmogorov-Smirnov tests had indicated that EP data were normally distributed, a multivariate analysis of variance (MANOVA) was performed, separately for latencies and amplitudes and for each bipolar recording site, respectively, using the factors “side” (left, right) and “group” (PD, CTRL) for statistical analysis of the ABVN-SEP, AEP and TN-SEP data. For the ABVN-SEP another analysis with “MoCA” as a covariate was performed, to control for effects related to cognitive impairment (MANCOVA). The MAN(C)OVA analysis of the ABVN-SEP data was also performed after having removed data over and above two standard deviations (SD, with and without “MoCA” as a covariate).

Subjects with pacemaker were excluded from the experiment; subjects with arrhythmias were excluded from the analysis. Explorative analysis of the HRV parameters revealed extreme outliers (lacking biological plausibility). Therefore, extreme outliers were excluded based on Tukey’s hinges (first quartile − 3 * interquartile range (IQR) and third quartile + 3 * IQR), visualized in boxplots [24]. Repeated measures ANOVA (rmANOVA) were performed for the HRV index and ln(LF/HF) ratio. Two-tailed t-tests were used for post hoc analysis. As ln(HF) and ln(LF) data were not normally distributed, non-parametric tests were used. Friedman test was applied to analyze the effect of CONDITION (rest, ABVN right, ABVN left) in each group separately. Group differences (PD, CTRL) were tested using Mann-Whitney for every condition separately.

Correlations between EP and HRV data and disease duration and UPDRS III were assessed using linear regression analysis (Pearson’s correlation).

Data were tested for nonsphericity using Mauchly’s test. In case of lack of sphericity, p values were corrected using the Greenhouse–Geisser correction. The false discovery rate correction (FDRC, [25]) was applied to correct for multiple comparisons. Effects were considered significant if p<0.05. All data are given as means ± SD. The statistics program used was SPSS for windows (version 20.0).

## Results

Patient and control groups were matched in terms of age (PD, 64.3±9.0 years; CTRL, 64.2±11.2 years; p = 0.961), sex distribution (m/f, both 29/21), but differed slightly in cognitive capacity as assessed by the MoCa test (PD, 24.6±3.1; CTRL, 25.8±2.7, p = 0.048). Disease stage was 2.3±0.7 on the Hoehn and Yahr scale, disease duration 6.4±4.5 years (range 1.0–20 years). Patients scored 20.9±7.2 in the motor part of the Unified Parkinson’s Disease Rating Scale (UPDRS III) and mean equivalent levodopa dose [26] was 707±480 mg.

**Fig 3:**
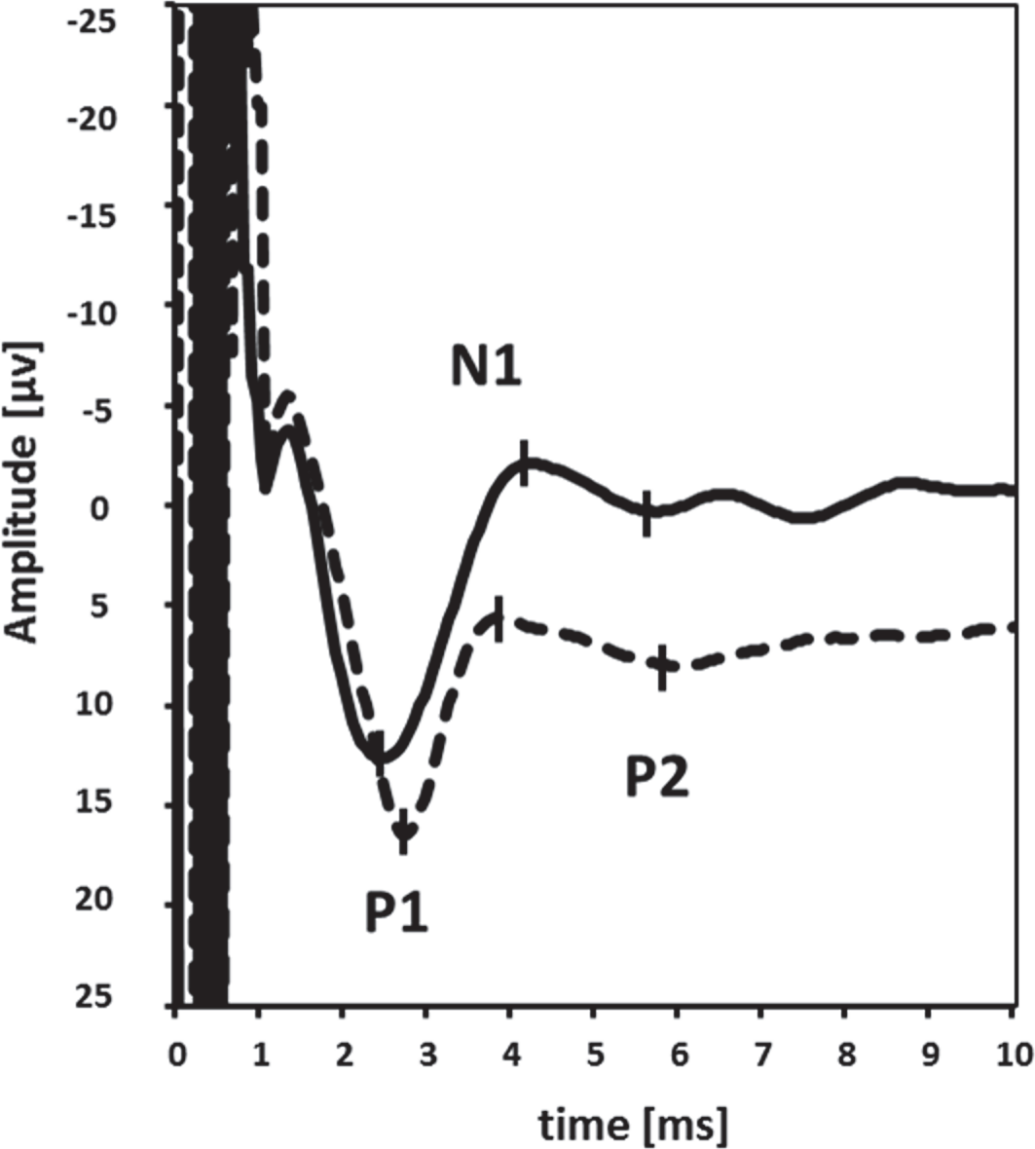
ABVN-SEP recorded at the electrode position A1-Cz after electric stimulation of the auricular branch of the left vagus nerve in a healthy control subject (dashed line) and a patient with Parkinson’s disease (continuous line). P1 first positive peak, N1 first negative peak, P2 second positive peak.

ABVN-SEP were present with identifiable peaks P1, N1, and P2 in all patients, except one (missing potentials after stimulation of the right ABVN), and all controls, except for missing potentials after stimulation of the left ABVN in 3 control subjects and of the right ABVN in 5 control subjects. The MANOVA identified a significant factor “group” only for two recording sites (Cz-A1/2; O1-T3/O2-T4) without correction for multiple comparisons each (four different recording sites). When the effect of cognition was removed by using MoCA as covariate, only one recording site (Cz-A1/2) remained significant. However, after FDRC, no significant group difference survived (Fig. 3, Table 1, S1 Table). Overall, differences between groups were numerically larger without consideration of cognition (MANOVA vs. MANCOVA), although the factor MoCA itself was not significant. When the analysis was performed after removal of outliers (data over and above 2 SD), again no differences were found between groups in any recording site. MANOVA and MANCOVA did not reveal any differences of peak-to-peak amplitudes between groups in any recording site (Table 1, S1 Table).

**Table 1:** Results of ABVN-SEP, AEP and TrigN-SEP.

		main effect				*PD*	*CTRL*
**ABVN-SEP**	*latency [ms]*	group	F(3,180) = 2.544	p = 0.058	P1	2.7 ± 0.5	2.8 ± 0.6
		side	F(3,180) = 0.792	p = 0.500	N1	4.5 ± 0.9	4.6 ± 1.0
		group * side	F(3,180) = 0.692	p = 0.558	P2	6.3 ± 1.2	6.1 ± 1.2
		MoCA	F(3,180) = 1.804	p = 0.148			
	*amplitude[μV]*	group	F(2,140) = 0.180	p = 0.835	P1-N1	11.8 ± 12.4	12.1 ± 11.3
		side	F(2,140) = 0.516	p = 0.598	N1-P2	6.8 ± 11.1	6.3 ± 6.4
		group * side	F(2,140) = 0.568	p = 0.568			
		MoCA	F(2,140) = 0.491	p = 0.613			
**AEP**	*latency [ms]*	group	F(5,189) = 0.310	p = 0.906	I	1.8 ± 0.2	1.8 ± 0.2
		side	F(5,189) = 0.316	p = 0.903	II	3.0 ± 0.2	3.0 ± 0.1
		group * side	F(5,189) = 0.445	p = 0.817	III	4.0 ± 0.2	4.0 ± 0.2
					IV	5.3 ± 0.2	5.2 ± 0.2
					V	5.9 ± 0.2	5.9 ± 0.2
**TrigN-SEP**	*latency [ms]*	group	F(3,193) = 0.659	p = 0.578	N13	13.9 ± 1.2	13.9 ± 1.0
		side	F(3,193) = 0.564	p = 0.639	P19	20.6 ± 1.6	20.4 ± 1.2
		group * side	F(3,193) = 1.882	p = 0.134	N27	28.1 ± 2.5	27.7 ± 1.5

Results of ABVN-SEP (recording site O1-T3/O2-T4), AEP (A1/2-Cz) and TrigN-SEP (C5/C6-Fz; mean ± SD). The main effect of the MAN(C)OVA for peak latencies and amplitudes is indicated. There were no group differences between groups in the other recordings-sites either after correction for multiple comparisons (ABVN-SEP). PD, patients with Parkinson’s disease; CTRL, healthy control subjects; ABVN-SEP, somatosensory evoked potentials following electric stimulation of the auricular branch of the vagus nerve; AEP, auditory evoked potentials; TrigN-SEP, somatosensory evoked potentials following electric stimulation of the trigeminal nerve.

MANOVA did not reveal any difference in AEP peak and interpeak latencies between PD patients and controls. TrigN-SEP latencies were not different between both groups either (Table 1).

No correlation of ABVN-SEP latencies and disease duration or UPDRS III was found in any condition (data not shown).

HRV index was lower in PD patients compared to healthy controls (Table 2). It was not altered by left or right ABVN stimulation in either group. These conclusions were supported by a significant rmANOVA that revealed a significant main effect of GROUP (PD, CTRL; F(1,31) = 10.184, p = 0.003), but no effect for the factor CONDITION (rest, ABVN right, ABVN left; F(2,62) = 1.513, p = 0.228) or for the interaction GROUP * CONDITION (F(2,62) = 0.851, p = 0.432).

**Table 2:** Results of HRV parameters at rest and following right and left ABVN stimulation.

	HRV index			ln(HF)			ln(LF)			ln(LF/HF)		
**condition**	*rest*	*ABVN r*	*ABVN l*	*rest*	*ABVN r*	*ABVN l*	*rest*	*ABVN r*	*ABVN l*	*rest*	*ABVN r*	*ABVN l*
**PD**	6.2 ± 0.9	6.1 ± 0.9	6.1 ± 0.9	0.97 ± 0.14	1.07 ± 0.16	1.00 ± 0.15	0.46 ± 0.07	0.49 ± 0.07	0.52 ± 0.08	0.55 ± 0.08	0.38 ± 0.06	0.48 ± 0.07
**CTRL**	7.9 ± 1.3	8.0 ± 1.3	7.5 ± 1.2	1.52 ± 0.24	1.70 ± 0.28	1.40 ± 0.22	1.02 ± 0.16	0.89 ± 0.15	0.77 ± 0.12	0.60 ± 0.09	0.48 ± 0.08	0.53 ± 0.08
**p-value**	0.001	0.001	0.007	0.015	0.022	0.038	0.006	0.005	0.051	0.483	0.070	0.467

Results of HRV parameters at rest and following right and left ABVN stimulation (mean ± s.e.m.). HRV index, number of all RR-intervals / maximal frequency; ln(HF), power spectral analysis in the high frequency spectrum (0.15–0.5 Hz); ln(LF), power spectral analysis in the low frequency spectrum (0.05–0.15 Hz); ln(LF/HF), low frequency/high frequency ratio; ABVN, auricular branch of the vagus nerve; PD, patients with Parkinson’s disease; CTRL, healthy control subjects; r, right; l, left.

Ln(LF) and ln(HF) were not influenced by ABVN stimulation in any group either (PD ln(HF) χ2(2) = 1.556, p = 0.459; CTRL ln(HF) χ2(2) = 2.381, p = 0.304; PD ln(LF) χ2(2) = 1.841, p = 0.398; CTRL ln(HF) χ2(2) = 1.614, p = 0.446). Ln(LF) and ln(HF) however, were lower in PD patients compared to healthy controls in all conditions (apart from ln(LF) after left ABVN stimulation, Table 2).

The ln(LF/HF) ratio did not differ between both groups either at rest and or during ABVN stimulation (Table 2). These conclusions were supported by a non-significant rmANOVA for the factor GROUP (F(1,28) = 2.196, p = 0.150) and the interaction GROUP * CONDITION (F(2,56) = 0.323, p = 0.725). However, rmANOVA was significant for CONDITION (F(2,56) = 4.048, p = 0.023). Post-hoc t-test revealed a significant decrease of ln(LF/HF) ratio during right ABVN stimulation in comparison to the resting condition across all subjects (p = 0.002), but not following left VN stimulation (p = 0.238).

HRV index and ln(LF/HF) ratio did not correlate with disease duration or UPDRS III. UPDRS III correlated negatively with ln(LF) (r = −0.369, p = 0.014) and ln(HF) (r = −0.291, p = 0.050) indicating a (weak) association between impairment in cardiac autonomic modulations and motor deficit.

## Discussion

Physiological examination of brainstem function by stimulation of the ABVN did not reveal abnormalities in this population of PD patients compared to age and sex matched healthy controls. ABVN-SEP did not discriminate between groups, and this was even more obvious when cognition was considered as a covariate. Furthermore, ln(LF/HF) ratio, a measure sensitive to cardiac parasympathetic tone, was modulated by ABVN stimulation, but similarly in PD patients and controls.

The present study has employed ABVN-SEP recording as a means of assessing VN function [9]. The ABVN runs via the superior jugular ganglion into the medulla oblongata and to the spinal trigeminal and solitary nucleus [27]. Anatomical specificity of this pathway is suggested by the fact that ABVN-SEP potentials are blocked by application of local anesthetics in the stimulation area [9]. The notion that the ABVN-SEP exhibit far-field potentials generated at the brainstem level was recently challenged, as ABVN-SEP were found to disappear during neuromuscular blockade [28]. However, in a functional MRI study, brainstem activation along vagal afferent pathways was seen following transcutaneous ABVN stimulation [29,30]. In addition, we and others [9] observed that ABVN-SEP were only evoked following electrical stimulation of the cutaneous representation of the ABVN, but not at the skin supplied by the trigeminal nerve or the posterior auricular nerve, where myogenic potentials should also be evoked. Thus, we believe ABVN-SEP to reflect neuronal activity within afferent brainstem pathways. According to Braak and co-workers [1] in PD neuronal loss starts in the dorsal motor, efferent nucleus of the VN. Visceroafferent nuclei of the solitary tract may also, at least in part, be affected [6], however, the efferent nucleus ambiguous and afferent somatosensory nuclei remain spared. Normal ABVN-SEP in PD may then reflect absence of degeneration in neurons processing somatosensory afferent information. The general integrity of sensory brainstem and cortical pathways is also supported by the fact that no abnormalities were found in AEP [31] (but see [32]) and TrigN-SEP (not to our knowledge examined in PD before), respectively. As we did not investigate early (brainstem) components of the TrigN-SEP described previously [33], the present data allow for only indirect conclusions regarding the explicit sensory trigeminal brainstem pathway.

Our results appear to be at variance with previous findings obtained in a small sample of PD patients which demonstrated partially prolonged ABVN-SEP latencies, after left ABVN stimulation [11]. Because in the present study, the sensitivity to detect ABVN-SEP changes was enhanced by additional recording sites (established for recording of far field potentials such as AEPs), methodological factors are unlikely to explain this discrepancy. Prolonged latencies of ABVN-SEP have been described in previous studies in patients with AD compared to healthy controls [13,34], where degeneration of the vagal nuclei complex, possibly including parts processing somatosensory afferents, is present early in the disease course [14]. It, therefore, is conceivable that prolonged ABVN-SEP latencies found previously in PD patients [11] may reflect the co-presence of Alzheimer and PD pathology in these patients. In the present study, the range of cognitive scores in PD patients was small, as all patients had normal or near-normal cognition. This, and variable co-expression of Alzheimer pathology in cognitively impaired PD may explain why cognition did not emerge as a significant factor in our analysis.

Afferent and efferent parts of the vagal nuclei complex are closely functionally interconnected [27,35]. Vomiting, cough or even syncope may be induced by mechanical stimulation of the external acoustic meatus ("Arnold’s reflex" [16,36]) and such modulation of the cardiac parasympathetic autonomic pathways is an emerging therapeutic approach for cardiac disorders [17,19,18,37]. Recently, Clancy and colleagues demonstrated that cardioautonomic function was shifted toward parasympathetic predominance following electrical stimulation of the ABVN [15]. We found ln(LF/HF) ratio to be reduced by ABVN stimulation in healthy controls. Because the ln(LF/HF) ratio is believed to mirror sympathovagal balance [22], this observation may suggest that parasympathetic activity is relatively enhanced over sympathetic activity by afferent cutaneous stimulation, a finding in line with human studies [15] and observations in animals [38] although neither ln(HF), a marker for sympathetic activity [22], nor ln(HF), a marker for parasympathetic activity [22], were markedly modulated by ABVN stimulation. Specificity of this finding is suggested by the fact that only right, but not left ABVN stimulation was capable of modulating ln(LF/HF) ratio. Cardiac parasympathetic innervation of the sinoatrial node (and, therefore, modulation of heart beat frequency) is mainly subserved by nerve fibers emanating from right brainstem vagus nuclei [4]. Importantly, we found that modulation of ln(LF/HF) ratio by right ABVN stimulation was fully retained in PD patients. This observation provides an indirect clue that vagal nuclei complex function may in fact not be severely functionally compromised in PD. Preserved capacity for the cutaneo-autonomic pathway may indicate intact function of the nucleus ambiguous which, as noted above, is relatively spared in PD [1,6]. Alternatively, as DMN is involved also in regulating cardiac activity [4,3], retained cutaneo-autonomic reflex function as indexed by ln(LF/HF) ratio may indicate less degeneration of DMN than previously thought. In line with the latter conclusion, some authors have argued that DMN is not severely affected by alpha-synuclein pathology in early disease stages of PD [39,40].

Despite retained cutaneo-autonomic modulation, PD patients had evidence of subclinical autonomic dysfunction as shown by a decrease of different HRV indices, in agreement with previous studies [41–43]. Changes of HRV in PD may at least in part be due to sympathetic cardiac denervation [44,43,45]. However, impairment of HRV did not correlate with postganglionic cardiac denervation as revealed by cardiac scintigraphy [45,43]. Intact ABVN-SEP and intact modulation of parasympathetic tone by ABVN-stimulation may then suggest that autonomic dysfunction either starts in the peripheral autonomic system [46], or is related to norepinephrine loss in the central sympathetic nervous system [47].

In conclusion, the present study has not provided evidence favoring malfunctioning of ABVN or cutaneo-autonomic reflex operation involving the ABVN in PD patients. The sensory part of the VN may not be functionally affected in PD.

## Supporting Information

S1 Table Results of ABVN-SEP at the recording site Fz-F3/F4, C3-F3/C4-F4, Cz-A1/A2). The main effect of the MANCOVA for peak latencies and amplitudes is indicated. There were no group differences between groups either after correction for multiple comparisons (ABVN-SEP). PD, patients with Parkinson’s disease; CTRL, healthy control subjects; ABVN-SEP, somatosensory evoked potentials following electric stimulation of the auricular branch of the vagus nerve.
(DOC)
