# Supplementary material for: Assessment of Brainstem Function with Auricular Branch of Vagus Nerve Stimulation in Parkinson’s Disease
Source: PLoS One. 2015 Apr 7;10(4):e0120786. doi: 10.1371/journal.pone.0120786 (PMC4388709; doi:10.1371/journal.pone.0120786)
Supplement: S1 Table — The main effect of the MANCOVA for peak latencies and amplitudes is indicated. There were no group differences between groups either after correction for multiple comparisons (ABVN-SEP). PD, patients with Parkinson’s disease; CTRL, healthy control subjects; ABVN-SEP, somatosensory evoked potentials following electric stimulation of the auricular branch of the vagus nerve. (DOC) [file pone.0120786.s001.doc]

|  |  | main effect | | |  | *PD* | *CTRL* |
| --- | --- | --- | --- | --- | --- | --- | --- |
| **Fz–F3** | *latency [ms]* | group | F(3,180) = 2.395 | p = 0.070 | P1 | 2.7 ± 0.5 | 2.7 ± 0.5 |
|  |  | side | F(3,180) = 0.166 | p = 0.919 | N1 | 4.9 ± 0.9 | 4.6 ± 0.9 |
|  |  | group * side | F(3,180) = 0.226 | p = 0.850 | P2 | 6.5 ± 1.2 | 6.1 ± 1.0 |
|  |  | MoCA | F(3,180) = 1.059 | p = 0.368 |  |  |  |
|  | *amplitude[µV]* | group | F(2,167) = 0.811 | p = 0.446 | P1-N1 | 3.1 ± 2.3 | 3.4 ± 2.6 |
|  |  | side | F(2,167) = 0.895 | p = 0.411 | N1-P2 | 1.8 ± 0.9 | 1.7 ± 1.1 |
|  |  | group * side | F(2,167) = 0.107 | p = 0.899 |  |  |  |
|  |  | MoCA | F(2,167) = 1.327 | p = 0.268 |  |  |  |
|  |  |  |  |  |  |  |  |
| **C3-F3** | *latency [ms]* | group | F(3,179) = 1.379 | p = 0.251 | P1 | 2.5 ± 0.4 | 2.6 ± 0.6 |
|  |  | side | F(3,179) = 0.080 | p = 0.971 | N1 | 4.4 ± 0.8 | 4.3 ± 0.8 |
|  |  | group * side | F(3,179) = 0.465 | p = 0.707 | P2 | 6.1 ± 1.1 | 5.9 ± 1.2 |
|  |  | MoCA | F(3,179) = 1.695 | p = 0.170 |  |  |  |
|  | *amplitude[µV]* | group | F(2,126) = 1.654 | p = 0.195 | P1-N1 | 3.0 ± 2.1 | 2.8 ± 2.3 |
|  |  | side | F(2,126) = 0.360 | p = 0.698 | N1-P2 | 1.8 ± 1.3 | 1.7 ± 1.1 |
|  |  | group * side | F(2,126) = 1.138 | p = 0.324 |  |  |  |
|  |  | MoCA | F(2,126) = 0.853 | p = 0.429 |  |  |  |
|  |  |  |  |  |  |  |  |
| **Cz-A1** | *latency [ms]* | group | F(3,180) = 3.299 | p = 0.022 | P1 | 2.6 ± 0.3 | 2.6 ± 0.5 |
|  |  | side | F(3,180) = 0.510 | p = 0.676 | N1 | 4.8 ± 0.7 | 4.6 ± 0.8 |
|  |  | group * side | F(3,180) = 0.961 | p = 0.412 | P2 | 6.5 ± 1.0 | 6.4 ± 1.1 |
|  |  | MoCA | F(3,180) = 3.691 | p = 0.013 |  |  |  |
|  | *amplitude[µV]* | group | F(2,150) = 0.717 | p = 0.490 | P1-N1 | 14.7 ± 11.5 | 13.6 ± 11.1 |
|  |  | side | F(2,150) = 0.641 | p = 0.528 | N1-P2 | 2.8 ± 2.2 | 3.3 ± 3.1 |
|  |  | group * side | F(2,150) = 0.641 | p = 0.184 |  |  |  |
|  |  | MoCA | F(2,150) = 1.131 | p = 0.325 |  |  |  |

**Table S1.** Results of ABVN-SEP at the recording site Fz-F3/F4, C3-F3/C4-F4, Cz-A1/A2). The main effect of the MANCOVA for peak latencies and amplitudes is indicated . There were no group differences between groups either after correction for multiple comparisons (ABVN-SEP). PD, patients with Parkinson’s disease; CTRL, healthy control subjects; ABVN-SEP, somatosensory evoked potentials following electric stimulation of the auricular branch of the vagus nerve.
